# PLIN1 suppresses glioma progression through regulating lipid metabolism

**DOI:** 10.1038/s41419-025-07347-z

**Published:** 2025-01-27

**Authors:** Kui Luo, Kai Zhuang, Hao Wu, Yuanbing Chen, Yi Liu, Fan Yang, Zhifei Wang

**Affiliations:** Department of Neurosurgery, The Third Xiangya Hospital, Central South University, Changsha, Hunan China

**Keywords:** CNS cancer, CNS cancer

## Abstract

Glioma is a common and destructive brain tumor, which is highly heterogeneous with poor prognosis. Developing diagnostic and prognostic markers to identify and treat glioma early would significantly improve the therapeutic outcomes. Here, we conducted RNA next-generation sequencing with 33 glioma samples and 15 normal brain samples. We found Perilipin 1 (PLIN1) downregulated in glioma and correlated with poorer outcome. Subsequent experiments revealed that up regulation of PLIN1 led to repressed cell growth and invasion in glioma. Moreover, overexpression of PLIN1 increased lipid accumulation in glioma cells, with increasing expression of lipid biosynthesis related genes and decreasing expression of lipolysis related genes. Mechanically, we revealed that the PI3K/AKT axis could regulate PLIN1 levels in glioma, that inhibition of the activity of PI3K/AKT axis could increase PLIN1 levels in glioma. In conclusion, the dysregulation PI3K/AKT axis led to PLIN1 downregulation and the following tumor proliferation, invasion and lipid metabolism reprogramming in glioma.

## Introduction

Glioma is by far the most frequently diagnosed malignant primary brain tumor in adults, which is highly heterogeneous with poor prognosis [1]. As the emerging of immunotherapy and targeted therapy, specific biomarkers are under evaluation in pre-clinical and clinical trials to guide effective precise treatment in glioma [2].

Metabolic reprogramming is a hallmark of cancer, that cancer cells could change metabolism to promote proliferation and survival. Lipid metabolic reprogramming, such as increased lipolysis, was seen in a variety of malignancies, promoting tumor progression [3, 4]. In glioma, lipid metabolism dysregulation has also been recognized as a vital trigger for tumor development [5]. For instance, hsa-miR-338-3p targeted mitochondrial 3-oxoacyl-ACP synthase to modulate fatty acid metabolism to repress glioma cell growth [6]. However, the mechanism of lipid metabolism in glioma progression is unclear so far. Investigating the function and mechanism of lipid metabolism axis in glioma would help develop more specific target to treat glioma.

The Perilipin (PLIN) family takes part in lipid metabolism modulation in tumor microenvironment to regulate the development of malignancies [7]. PLIN1 belongs to the PLIN family, and acts as an important regulator of lipid droplets (LDs), which could protect LDs from hydrolysis by lipases [8, 9]. It is reported that dysregulation of PLIN1 is involved in carcinogenesis. In breast cancer, PLIN1 is identified as a tumor suppressor, which is lowly expressed and may act as marker or therapeutic target [10–12]. In hepatocellular carcinoma (HCC), PLIN1 is also negatively expressed in HCC cell LDs [13]. Besides, PLIN1 was highly expressed in most liposarcomas, which could be a promising specific diagnostic marker [14–16]. But the possible functions and mechanism of PLIN1 in glioma is still elusive.

Here in this study, we conducted RNA next-generation sequencing with 33 glioma samples and 15 brain normal samples to discover that PLIN1 was downregulated in glioma. Further experiments revealed that PLIN1 up regulation led to repressed glioma growth and invasion. Moreover, PLIN1 could regulate lipid metabolism in glioma. Mechanically, KEGG pathway analysis and GO analysis demonstrated the dysregulation of PI3K/AKT axis in glioma, which could lead to the downregulation of PLIN1 and the following tumor proliferation and invasion in glioma.

## Materials and methods

### Tissue specimens

For microarray analysis, we collected 33 tumor samples and 15 brain normal samples during surgery from glioma patients in the Third Xiangya Hospital of Central South University. Moreover, for qRT-PCR confirmation, 30 paired glioma tissues along with normal adjacent tissues were harvested. The Ethics Committee of the Third Xiangya Hospital of Central South University approved this study, and 1964 Helsinki declaration was followed. Patients involved had provided informed consents.

### Collection and analysis of data

The lower grade glioma and glioblastoma (GBMLGG) cohort from TCGA including 5 normal samples, 670 primary tumor samples as well as 27 recurrent tumor samples, was downloaded from UCSC Xena project. We calculated the overall survival (OS) in lower grade glioma, determined with cutoff 25% lower percentile and 25% upper percentile of PLIN1 levels with the OncoLnc Kaplan Plot (http://www.oncolnc.org).

### Cell lines, culture and transfection

All the cell lines, including adipocyte 3T3-L1, normal glial cell (HEB) along with glioma cells (U87, U251, SF126, SF767, T98, A712 and SHG-44) were bought from ATCC (USA) and cultured in accordance with the supplier’s instructions. The cells were authenticated and tested for mycoplasma contamination by DNA fingerprinting.

U87, U251 and SF767 cells were transfected by PLIN1 overexpression vector or control vector (NC), while SF126, T98 and SHG-44 cells were transfected by siRNA against PLIN1 or control (si-NC) by Lipofectamine 3000 (Invitrogen, USA).

### qRT-PCR

RNAs were extracted with TRIzol and mRNA relative expression was detected with SYBR® Premix Ex Taq™ (Takara, China) and determined with 2^-△△CT^. The primers were bought from Invitrogen: PLIN1 forward, 5’-CCATGTCCCTATCAGATGCCC-3’, reverse, 5’-CTGGTGGGTTGTCGATGTC-3’; GAPDH forward, 5’-CTGGGCTACACTGAGCACC-3’, reverse, 5’-AAGTGGTCGTTGAGGGCAATG-3’.

### CCK-8 assay

U87, U251 and SF767 (3*10^3^ cells/well) were planted then treated by PLIN1 overexpression vector or control vector, while SF126, T98 and SHG-44 cells (3*10^3^ cells/well) were treated by si-PLIN1 or control (si-NC). CCK-8 reagent (Beyotime, China) was added 24, 48 or 72 h later and cultured for another hour before detection of 490 nm absorbance.

### Colony formation assay

U87, U251 and SF767 (1*10^3^ cells/well) were planted then treated by PLIN1 overexpression vector or control vector, while SF126, T98 and SHG-44 cells (1*10^3^ cells/well) were treated by sh-PLIN1 or control (NC). Two weeks later, we fixed and stained the colonies with 4% paraformaldehyde and 0.5% crystal violet respectively for 20 min each. Finally, the colony numbers were counted.

### Transwell assay

Briefly, U87, U251 and SF767 cells (3*10^4^ cells/well) were transfected with PLIN1 overexpression vector or control vector, while SF126, T98 and SHG-44 cells (3*10^4^ cells/well) were treated by si-PLIN1 or control (si-NC), then re-suspended and introduced to the upper chambers (FBS-free medium) and 20% FBS medium was introduced to the lower chambers (BD Biosciences). The upper compartment’s Matrigel was removed 48 h later, while the infiltrating cells adherent to the bottom were fixed with Methanol for 10 min. The invasive cells were photographed and enumerated after being stained with crystal violet (2%) for 20 min.

### Mouse xenograft model construction and immunohistochemistry (IHC) analysis

U87 treated with PLIN1 overexpressing vector or SHG-44 treated with sh-PLIN1 vector (2*10^6^) were subcutaneously injected to the dorsal flanks of 4-week-old male BALB/c nude mice (six mice per group). Untreated U87 or SHG-44 cells were used as controls. The xenograft tumors were excised under anesthesia after 28 days, and the tumor weights were measured. Then, the tumors were submitted to IHC staining with anti-PLIN1 antibody (1:100, #DF7602, Affinity, USA) or anti-Ki67 antibody (1:100, #AF0198, Affinity). Animal studies were approved by the Third Xiangya Hospital of Central South University Institutional Animal Care and Use Committee. A sample size of at least five mice per group was estimated to obtain a power of 0.9 and an effect size of 0.95. No randomization was used. No blinding was done.

### Oil red O staining

Briefly, U87, U251 as well as SF767 cells (2*10^5^ cells/well) were planted then treated by PLIN1 overexpression vector or control vector, while SF126, T98 and SHG-44 cells (2*10^5^ cells/well) were treated by si-PLIN1 or control (si-NC). After fixing for 10 min by 4% paraformaldehyde, cells were washed by PBS and stained by Oil red O reagent (Beyotime) at room temperature to detect the lipid droplet (LD) levels for 20 min. At last, the cells were washed by PBS and photographed under a light microscope. The lipid contents of cells are quantified with ImageJ software. Briefly, the image was adjusted to 8-bit then the mean gray value of intrested area was measured.

### Detection of fatty acid metabolism

Briefly, U87, U251 as well as SF767 cells (3*10^6^ cells/well) were planted then treated by PLIN1 overexpression vector or control vector, while SF126, T98 and SHG-44 cells (3*10^6^ cells/well) were treated by si-PLIN1 or control (si-NC). The levels of triglyceride (TG) as well as free fatty acid (FFA) of cells were detected with Triglyceride Assay Kit (Abcam, USA) and Free Fatty Acid Quantification Kit (Abcam) according to the instructions of the manuals.

### Western blotting

Proteins were isolated by RIPA and PMSF, and separated via 10% SDS-PAGE. Then protein was transferred to PVDF membranes then blocked for an hour with 5% skim milk powder at room temperature before incubated with antibodies, included PLIN1 (1:1000, #DF7602), adipose triglyceride lipase (ATGL) (1:1000, #DF7756), hormone-sensitive lipase (HSL) (1:500, #AF6403), fatty acid synthase (FASN) (1:1000, #DF6106,), diacylglycerol acyltransferase-1 (DGAT-1) (1:1000, #DF13368), DGAT-2 (1:2000, #DF9442), PI3K (1:500, #AF4669), p-PI3K(Tyr607) (1:500, #AF3241), AKT (1:500, #AF6261), p-AKT(Ser473) (1:500, #AF0016), GAPDH (1:10000, #AF7021) at 4 °C overnight. Then, the membranes were incubated by secondary antibodies (1:5000, #S0001). All the above antibodies were bought from Affinity. At last, the membranes were treated with enhanced chemiluminescence reagent (Yeasen) and relative grayscale was quantified by ImageJ software. Full and uncropped western blots were shown in Supplemental Material.

### Statistical analysis

Statistical analysis was performed with GraphPad prism 9.0. Experiment was conducted thrice and result was showed as mean ± SD of three repeated experiments Significance was confirmed when *P* ≤ 0.05 by student’s *t* test.

## Results

### PLIN1 is downregulated and correlated with worse outcome of glioma

**Fig. 1:**
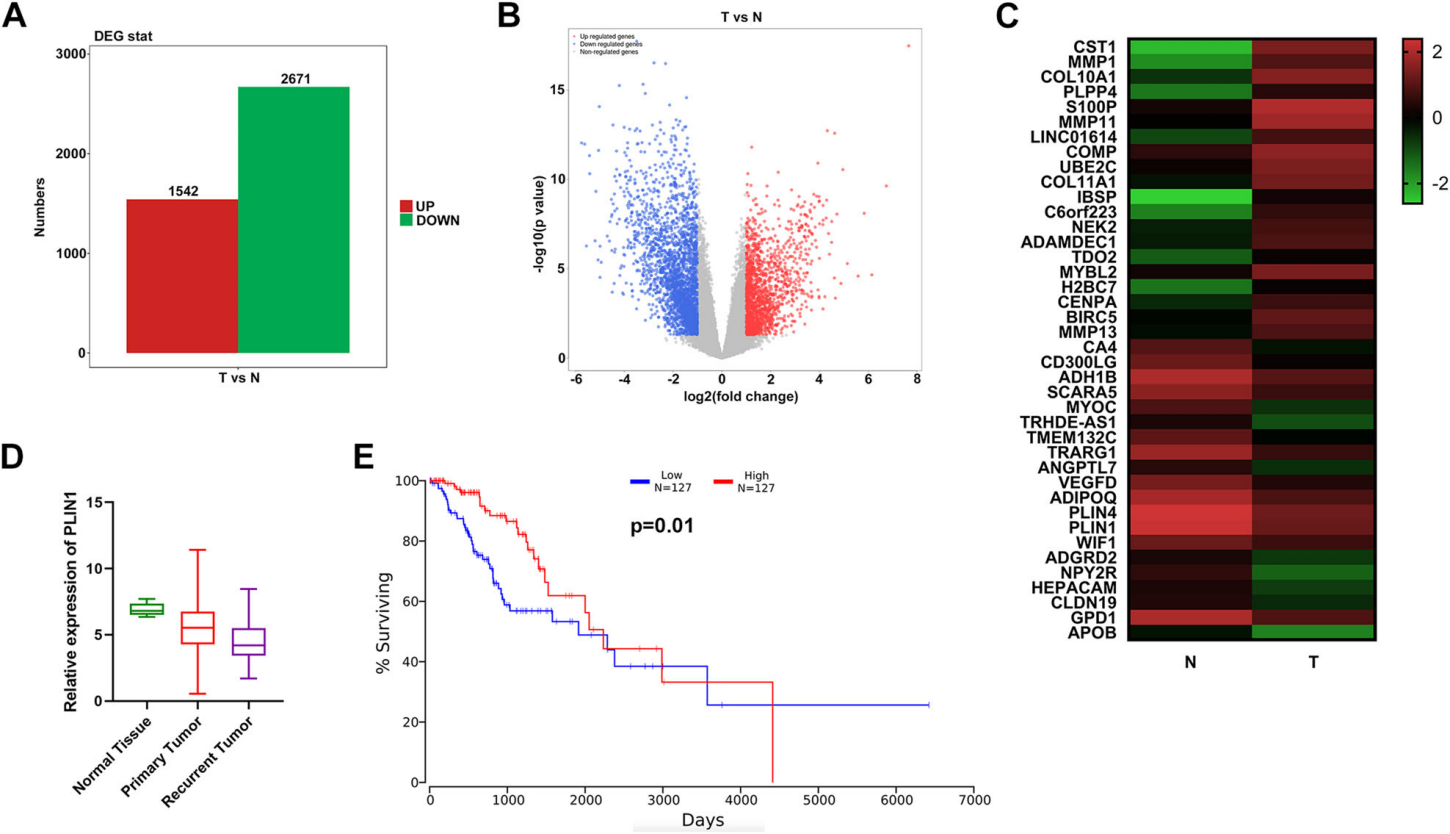
PLIN1 is downregulated and correlated with worse outcome of glioma. **A** Differentially expressed genes in 33 glioma tissues (T) compared with 15 normal brain tissues (N). Red: up regulated; Green: down regulated. Fold change > 2 and p < 0.05. **B** Volcano plots showing genes differentially expressed in 33 glioma tissues (T) compared with 15 normal brain tissues (N). Red: upregulated; Blue: downregulated. Fold change > 2 and p < 0.05. **C** Hierarchical clustering showing the top 20 upregulated or downregulated genes in glioma samples (T) compared with normal samples (N). Red: up regulated; Green: down regulated. **D** PLIN1 levels in lower grade glioma and glioblastoma (GBMLGG) dataset from TCGA including 5 normal samples, 670 primary tumor samples as well as 27 recurrent tumor samples were shown. **E** The OS curve of PLIN1 in lower grade glioma downloaded from the OncoLnc Kaplan Plot website was shown.

To investigate the molecular alterations in glioma, we conducted RNA next-generation sequencing with 33 glioma samples and 15 brain normal samples. Compared with normal samples, 1542 genes were elevated and 2671 genes were reduced with fold change > 2 and p < 0.05 in glioma tissues (Fig. 1A, B). Hierarchical clustering in Fig. 1C presented the top 20 upregulated or downregulated genes in glioma tissues compared to normal tissues. Among these candidate genes, we noticed PLIN1, which is recognized as a tumor suppressor in a variety of malignancies. But the possible biological function as well as mechanism of the PLIN1 in glioma is elusive so far. Hierarchical clustering in Fig. 1C indicated that PLIN1 was downregulated in glioma tissues. Moreover, we downloaded a lower grade glioma and glioblastoma (GBMLGG) dataset from TCGA and found out PLIN1 expressed lowly in primary tumor samples, and even lower in recurrent tumor tissues (Fig. 1D). On the other hand, we explored the effect of PLIN1 level on glioma outcome and found that low PLIN1 level was correlated with shortened OS of lower grade glioma (Fig. 1E). Taken together, the above findings indicated that PLIN1 expressed lowly in glioma and associated with poorer outcome.

### PLIN1 suppresses proliferation and invasion of glioma

**Fig. 2:**
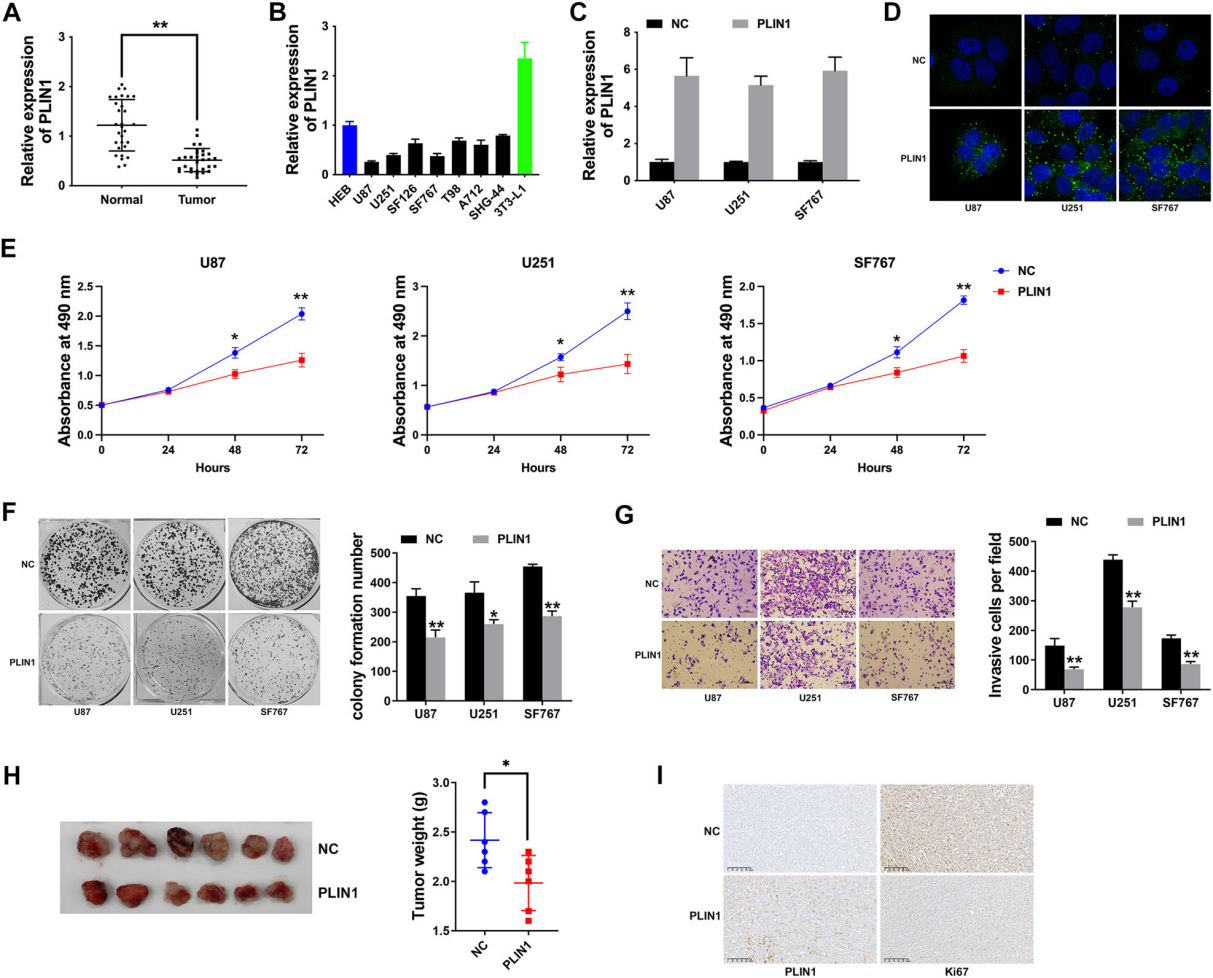
PLIN1 suppresses proliferation and invasion of glioma. **A** PLIN1 levels in 30 paired glioma samples and normal adjacent samples by qRT-PCR. **B** PLIN1 levels in glioma cells were detected by qRT-PCR. **C** PLIN1 overexpression in glioma cells was confirmed by qRT-PCR. **D** The intracellular localization and the overexpression of PLIN1 in glioma cells were detected by Immunofluorescence staining. **E** CCK-8 assay after PLIN1 overexpression. **F** Colony formation assay after PLIN1 upregulation. Left: represent image of colony formation assay; Right: colony formation number was quantified by ImageJ software. **G** Transwell assay after PLIN1 upregulation. Left: represent image of Transwell assay; Right: invasive cell number was quantified. **H** Glioma xenograft models were constructed with U87-PLIN1 overexpressing cell or non-treated U87 cell as control. Left: represents image of xenograft tumors. Right: the tumor weights were measured. **I** The expression of PLIN1 and Ki67 in xenograft tumors were detected by IHC staining. *P < 0.05, **P < 0.01. The experiment was repeated three times independently.

To confirm PLIN1 level in glioma, we detected PLIN1 expression in 30 paired glioma samples and adjacent normal samples. Figure 2A showed that PLIN1 was indeed reduced in glioma samples compared to normal samples. Moreover, Fig. 2B presented that PLIN1 also expressed lowly in glioma cells, particularly in U87, U251 and SF767. Thus, we applied overexpression vector to increase the level of PLIN1 in these three cells in order to explore the role of PLIN1 in glioma (Fig. 2C). Immunofluorescence staining of PLIN1 in Fig. 2D confirmed the intracellular localization and the overexpression of PLIN1 in glioma cells. CCK-8 assay revealed that up regulation of PLIN1 suppressed glioma proliferation (Fig. 2E). Besides, PLIN1 overexpression suppressed glioma cell colony formation ability (Fig. 2F). Moreover, up regulation of PLIN1 suppressed glioma cell invasion, presented by Transwell assay in Fig. 2G. To deeper investigate the impact of PLIN1 in vivo, mice xenograft models were constructed. And PLIN1 overexpression notably suppressed tumor growth in vivo (Fig. 2H). Moreover, overexpression of PLIN1 reduced the expression of Ki67 in tumors (Fig. 2I). The above findings revealed the fact that PLIN1 could suppress the proliferation and invasion of glioma.

### Inhibition of PLIN1 enhances proliferation and invasion of glioma

**Fig. 3:**
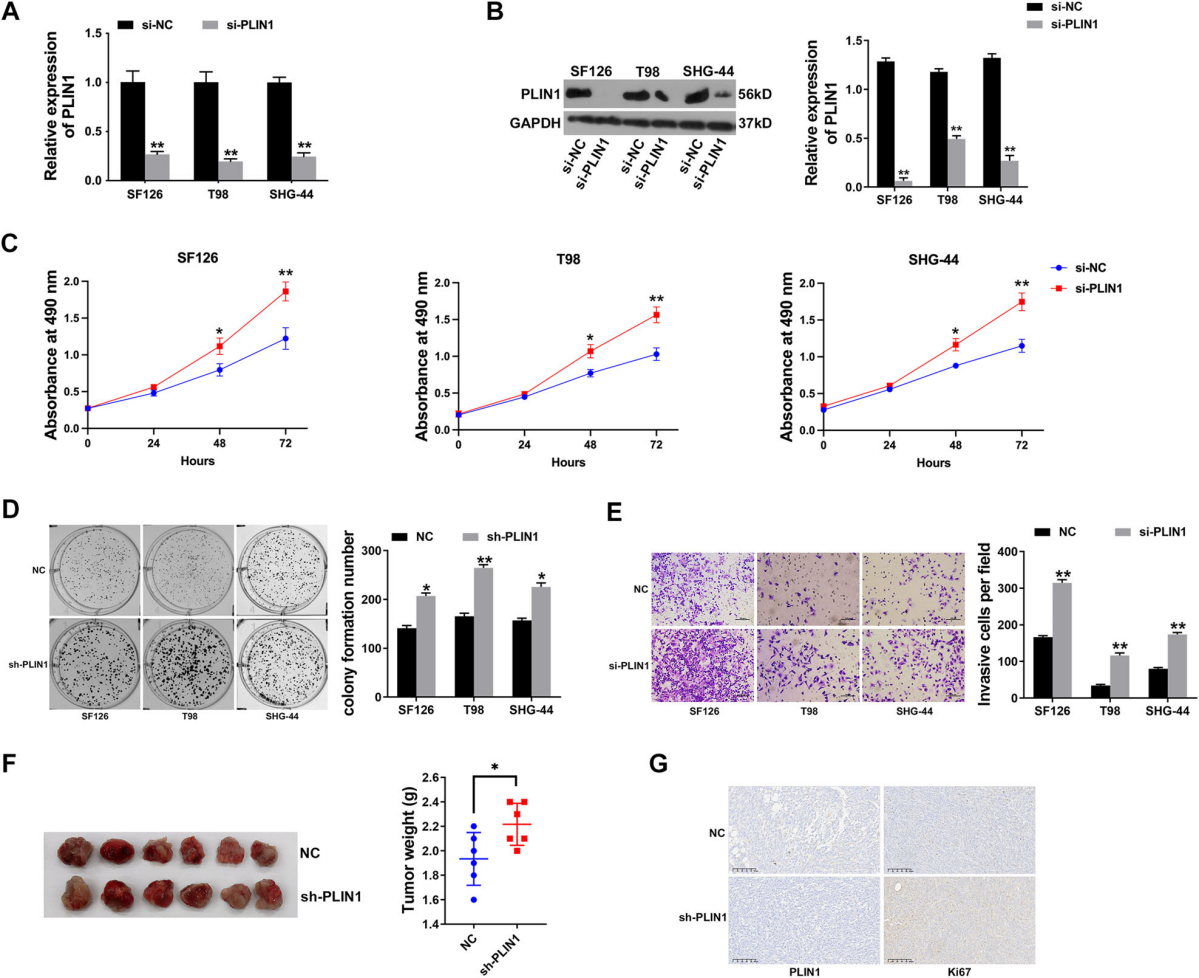
Inhibition of PLIN1 enhances proliferation and invasion of glioma. **A** Inhibition of PLIN1 in glioma cells was confirmed by qRT-PCR. **B** Inhibition of PLIN1 in glioma cells was confirmed by western blotting. And the western blotting bands were quantified with ImageJ software (Right). **C** CCK-8 assay after PLIN1 inhibition. **D** Colony formation assay after PLIN1 inhibition. Left: represent image of colony formation assay; Right: colony formation number was quantified by ImageJ software. **E** Transwell assay after PLIN1 inhibition. Left: represent image of Transwell assay; Right: invasive cell number was quantified. **F** Glioma xenograft models were constructed with SHG-44-PLIN1 knockdown cell or non-treated SHG-44 cell as control. Left: represent image of xenograft tumors. Right: the tumor weights were measured. **G** The expression of PLIN1 and Ki67 in xenograft tumors were detected by IHC staining. *P < 0.05, **P < 0.01. The experiment was repeated three times independently.

To better explore the impact of PLIN1 in glioma, we applied silencing vector to reduce the level of PLIN1 in SF126, T98 and SHG-44 cells (Fig. 3A, B). CCK-8 assay revealed that inhibition of PLIN1 enhanced glioma proliferation (Fig. 3C). Besides, PLIN1 inhibition increased glioma cell colony formation ability (Fig. 3D). Moreover, inhibition of PLIN1 promoted glioma cell invasion (Fig. 3E). Besides, mice xenograft models were constructed to investigate the role of silencing PLIN1 in glioma in vivo. And PLIN1 inhibition notably enhanced tumor growth in vivo (Fig. 3F). Moreover, inhibition of PLIN1 increased the expression of Ki67 in tumors (Fig. 3G). The above findings revealed that inhibition of PLIN1 in glioma would enhance cell proliferation and invasion.

### PLIN1 suppresses glioma progression through regulating lipid metabolism

**Fig. 4:**
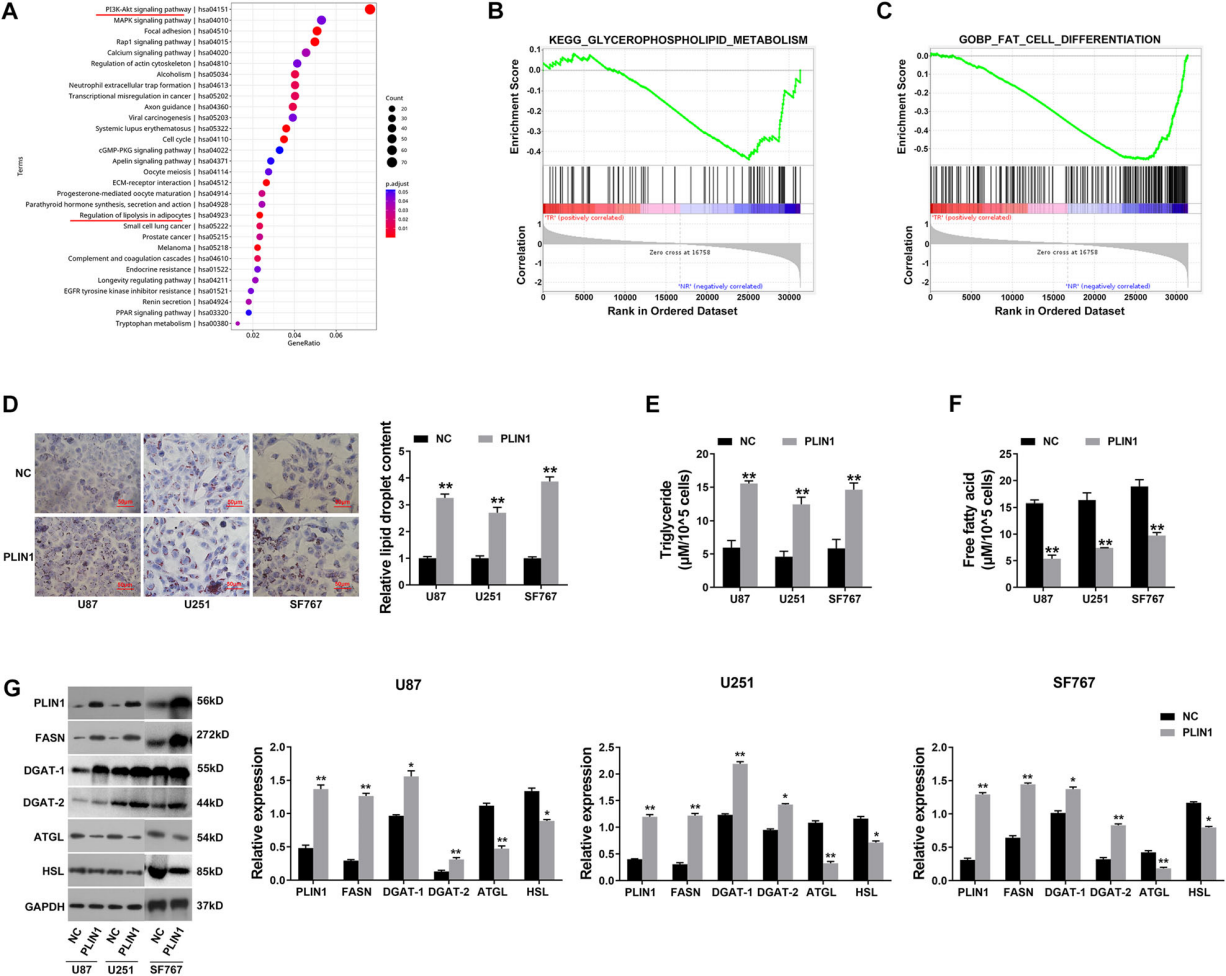
PLIN1 suppresses glioma progression through regulating lipid metabolism. **A** KEGG analysis in the above 33 glioma samples and 15 brain normal samples was shown. **B** GSEA analysis presented the relationship between glycerophospholipid metabolism and glioma tumorigenesis. **C** GSEA analysis presented the relationship between fat cell differentiation and glioma tumorigenesis. **D** Oil red O staining conducted to detect intracellular lipid contents after PLIN1 overexpression in glioma cell lines (Left). Intracellular lipid droplet contents were quantified by ImageJ software (Right). **E** The intracellular TG levels were detected after PLIN1 overexpression. **F** The intracellular FFA levels were detected after PLIN1 overexpression. **G** Lipid metabolism-related genes levels were determined by western blotting. The western blotting bands were quantified with ImageJ software (Right). *P < 0.05, **P < 0.01. The experiment was repeated three times independently.

Recently, lipid metabolism is getting more and more attention in cancer research. Enhanced lipogenesis is vital to cancer proliferation [17]. PLIN1 is involved in lipid metabolism modulation in tumor microenvironment and is correlated with tumor development as well as metastasis [18]. But the function and mechanism of PLIN1 in glioma lipid metabolism is still unclear nowadays. Therefore, KEGG enrichment analysis was applied to explore genes that differentially regulated in the above 33 glioma samples and 15 brain normal samples. Figure 4A showed the regulation of lipolysis in adipocytes was closely related with glioma tumorigenesis. GSEA analysis also claimed the association of glycerophospholipid metabolism, fat cell differentiation and glioma tumorigenesis (Fig. 4B, C). Thus, we continued to find out the function of PLIN1 in glioma lipid metabolism. The results showed that overexpression of PLIN1 increased lipid accumulation in glioma cells, presented by Oil Red O staining in Fig. 4D. Moreover, overexpression of PLIN1 increased the intracellular levels of TG in glioma cells, but decreased the levels of FFA (Fig. 4E, F). Increased lipid biosynthesis or decreased lipolysis could cause elevation of intracellular lipid content [19]. Therefore, we detected the expressions of key genes in lipid metabolism in order to investigate the underlying mechanism. We found PLIN1 up regulation increased the expression of lipid biosynthesis related genes FASN, DGAT-1 and DGAT-2, but reduced lipolysis related genes ATGL and HSL (Fig. 4G). The above results revealed that PLIN1 could suppress glioma progression partly through regulating lipid metabolism.

### The PI3K/AKT axis regulates PLIN1 expression in glioma

**Fig. 5:**
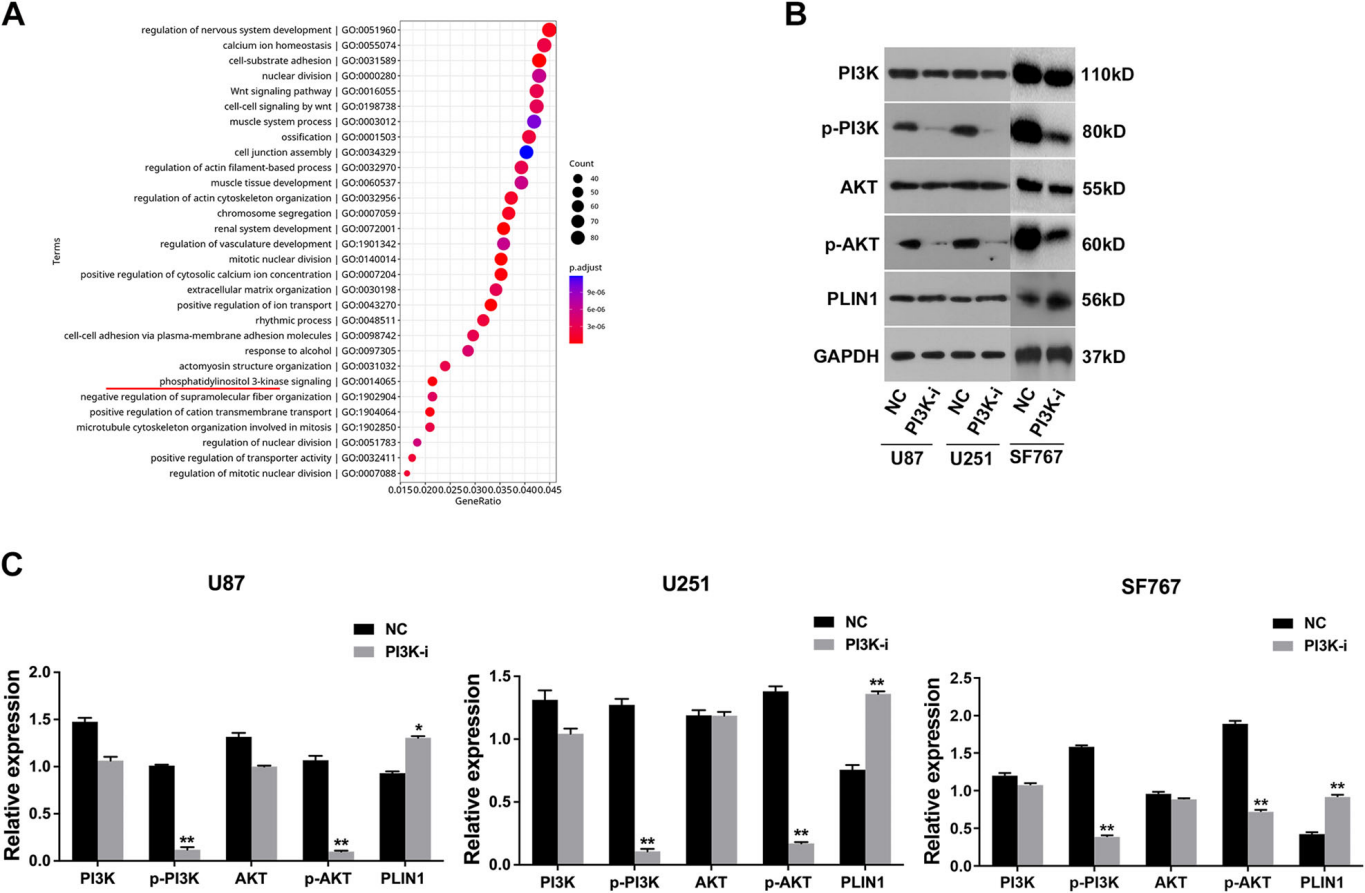
The PI3K/AKT axis regulates PLIN1 expression in glioma. **A** GO analysis in the above 33 glioma samples and 15 brain normal samples was shown. **B** Western blotting was performed after PI3K inhibition in glioma cell lines. **C** The western blotting result was quantified with ImageJ software. *P < 0.05, **P < 0.01. The experiment was repeated three times independently.

Next, we continued to explore the mechanism of PLIN1 downregulation in glioma. Both the KEGG (Fig. 4A) and the GO analysis (Fig. 5A) showed that the PI3K axis was related with glioma tumorigenesis. Therefore, we focused on the potential regulation of PI3K signaling pathway in PLIN1 expression in glioma. Using PI3K inhibitor LY294002, we successfully inhibited the PI3K signaling activity, demonstrated by decreased p-PI3K and p-AKT expression (Figs. 5B, 4C). As a result, the expression levels of PLIN1 were increased after inhibiting the PI3K/AKT axis. Thus, we concluded that PI3K/AKT axis dysregulation led to PLIN1 downregulation in glioma.

### The PI3K/AKT axis regulates glioma progression through PLIN1

**Fig. 6:**
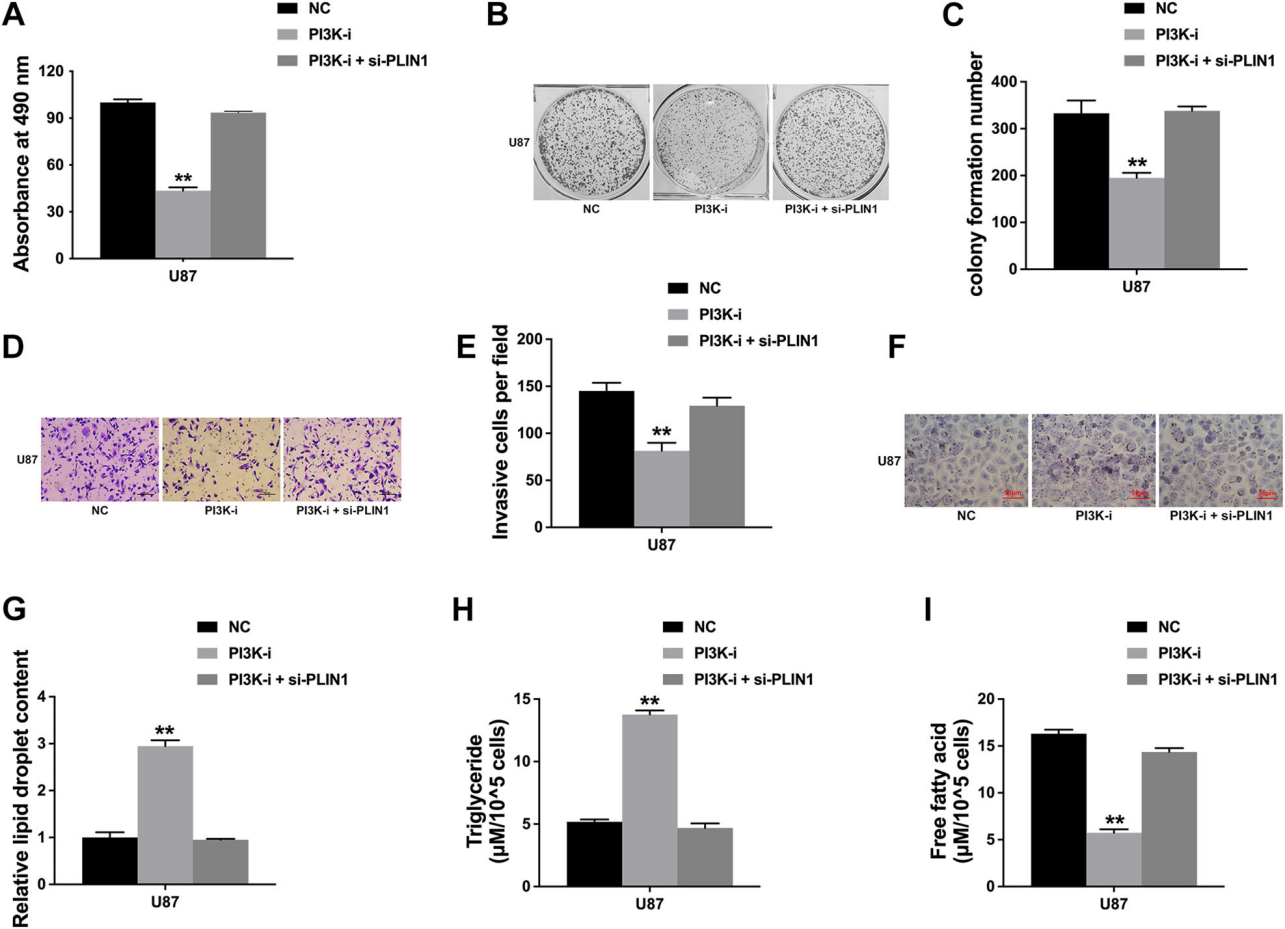
The PI3K/AKT axis regulates glioma progression through PLIN1. **A** CCK-8 assay was performed after cell were treated with PI3K inhibitor LY294002, LY294002 plus si-PLIN1 or control. **B** Colony formation assay was performed after cell were treated with LY294002, LY294002 plus si-PLIN1 or control. **C** Colony formation number was quantified by ImageJ software. **D** Transwell assay was performed after cell were treated with LY294002, LY294002 plus si-PLIN1 or control. **E** Invasive cell number was quantified by ImageJ software. **F** Oil red O staining conducted to detect intracellular lipid contents after cell were treated with LY294002, LY294002 plus si-PLIN1 or control. **G** Intracellular lipid droplet contents were quantified by ImageJ software. **H** The intracellular TG levels were detected after cell were treated with LY294002, LY294002 plus si-PLIN1 or control. **I** The intracellular FFA levels were detected after cell were treated with LY294002, LY294002 plus si-PLIN1 or control. **P < 0.01. The experiment was repeated three times independently.

We continued to explore the function of the PI3K/AKT axis in glioma progression. CCK-8 assay revealed that PI3K inhibition suppressed glioma cell proliferation, while adding of si-PLIN1 reversed the effect of PI3K inhibitor (Fig. 6A). Besides, inhibition of PI3K suppressed glioma cell colony formation ability, which was also reversed by si-PLIN1 (Fig. 6B, C). Moreover, Transwell assay showed that inhibition of PI3K suppressed glioma cell invasion and si-PLIN1 could reversed the effect of PI3K inhibitor (Fig. 6D, E). Oil Red O staining assay revealed that PI3K inhibition increased lipid accumulation in glioma cells, while adding of si-PLIN1 reversed the effect of PI3K inhibitor (Fig. 6F, G). Moreover, inhibition of PI3K increased the intracellular levels of TG, but reduced FFA levels, which was also reversed by si-PLIN1 (Fig. 6H, I). Thus, we concluded that PI3K/AKT axis dysregulation led to PLIN1 downregulation and the following tumor proliferation, invasion and lipid metabolism reprogramming in glioma.

## Discussion

Glioma is a common and destructive brain tumor, leading to a great burden to public health [20]. Developing diagnostic and prognostic markers to identify and treat glioma early would significantly improve the therapeutic outcomes [21]. Here, we conducted RNA next-generation sequencing with 33 glioma samples and 15 brain normal samples to find that PLIN1 was downregulated in glioma.

PLIN1 is recognized as a tumor suppressing gene in multiple malignancies, such as HCC [22, 23]. Meanwhile, the level of PLIN1 is reduced in glioblastoma multiforme and patients with elevated PLIN1 levels are associated with better prognosis in low-grade glioma [24]. Consistent with the above research, we discovered PLIN1 expressed lowly in primary glioma tissues, even lower in recurrent glioma tissues. Moreover, low PLIN1 expression in glioma was correlated with worse outcome. We also noticed that PLIN4 was also downregulated in glioma. However, when we explored the effect of PLIN4 on glioma prognosis with the OncoLnc Kaplan Plot (http://www.oncolnc.org), we found that PLIN4 level was not correlated with the outcome of glioma. Therefore, we ignored PLIN4 and just focused on PLIN1 in this study. Taken together, the above findings indicated that PLIN1 was downregulated in glioma and correlated with poorer outcome, indicating that PLIN1 might act as a promising biomarker in glioma diagnosis and prognosis.

Metabolism reprogramming in tumor cells and tumor microenvironment supports the progression of tumors, which is a potential treatment target for tumor therapy [25, 26]. But the underlying metabolic mechanism in glioma progression is unclear. Deregulation of lipid metabolism is common in cancers, which is a hot topic in cancer research [27]. For example, 15-lipoxygenase-1 (15-LOX) promoted the progression of glioblastoma. And inhibition of 15-LOX suppressed the proliferation and metastasis of glioblastoma [28]. Moreover, ACSVL3, a fatty acid metabolism enzyme, promoted the progression of glioblastoma cells partly via regulating cellular sphingolipid metabolism [29]. In glioma, lipid metabolism related genes are associated with clinicopathological features and immune checkpoint genes [30]. But the function of lipid metabolism is not well explored in glioma carcinogenesis as well as progression. Identifying tumor-specific molecules that could serve as diagnostic markers or therapeutic targets would help improve the outcome of glioma patients.

PLIN1 belongs to the PLIN family that involved in tumor lipid metabolism modulation [31]. PLIN1 regulates lipid synthesis as well as lipolysis to maintain lipid metabolism homeostasis [32]. However, the role and molecular mechanism of PLIN1 in glioma lipid metabolism is uncertain nowadays. Here, we claimed that overexpression of PLIN1 repressed growth and invasion of glioma cells. Moreover, overexpression of PLIN1 increased lipid accumulation in glioma cells, with increasing expression of lipid biosynthesis related genes and decreasing expression of lipolysis related genes. Taken together, the above findings indicated that PLIN1 was involved in glioma lipid metabolism, proliferation and invasion.

To investigate the mechanism of PLIN1 downregulation in glioma, we conducted both the KEGG and the GO analysis. The results revealed the involvement of the PI3K/AKT axis in glioma tumorigenesis. It is reported that the PI3K/AKT signaling could promote tumor progression in glioma [33]. For instance, the PI3K/AKT signaling activation could induce SLC7A11 expression, leading to treatment resistance in glioma [34]. Moreover, the PI3K/AKT signaling has been recognized engaged in lipid metabolism in multiple malignancies, such as in non-small cell lung cancer (NSCLC) [35], HCC [36] and ovarian cancer [37]. However, the impact of the PI3K/AKT axis on glioma lipid metabolism is unclear so far. Here, we found that PI3K/AKT axis activity inhibition could increase PLIN1 levels in glioma. We concluded that the PI3K/AKT axis dysregulation led to PLIN1 downregulation and the following tumor growth and invasion of glioma.

Taken together, this study claimed that PLIN1 acted as a tumor suppressor in glioma development and progression. PLIN1 could act as a promising biomarker in glioma diagnosis, prognosis and treatment. Nowadays, the prospect of targeting lipid metabolism in malignancies has been acknowledged. However, there is still no drug targeted PLIN1 that has gained clinical approval. Further in-depth study is warrant to develop PLIN1-targeted agents and help put PLIN1 into clinical usage for malignancies.

## Supplementary information

Original western blots

## Data Availability

All data and materials used in this study are available from the corresponding author upon reasonable request.
