# Supplementary figures and images for: PLIN1 suppresses glioma progression through regulating lipid metabolism
Source: Cell Death Dis. 2025 Jan 27;16(1):48. doi: 10.1038/s41419-025-07347-z (PMC11772837; doi:10.1038/s41419-025-07347-z)

F3B


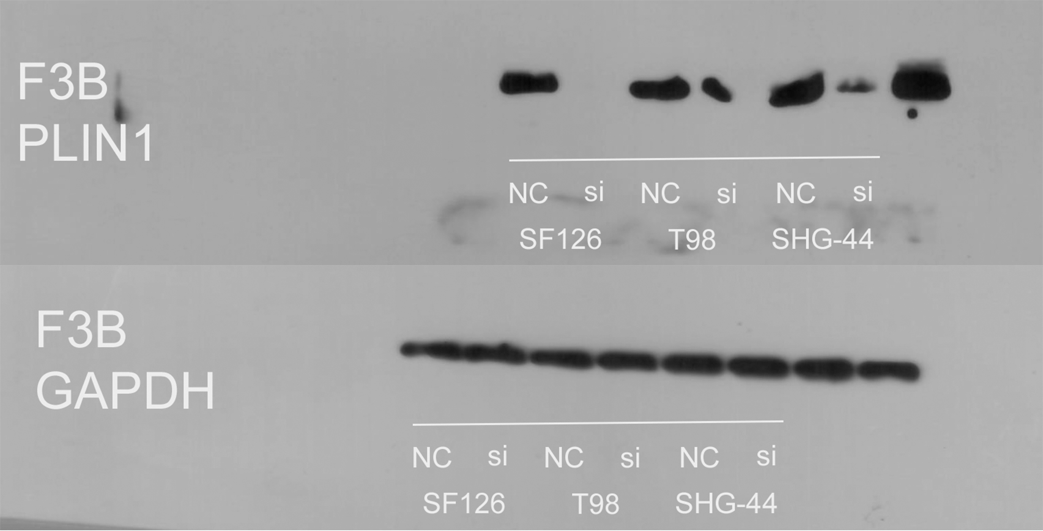


F4G


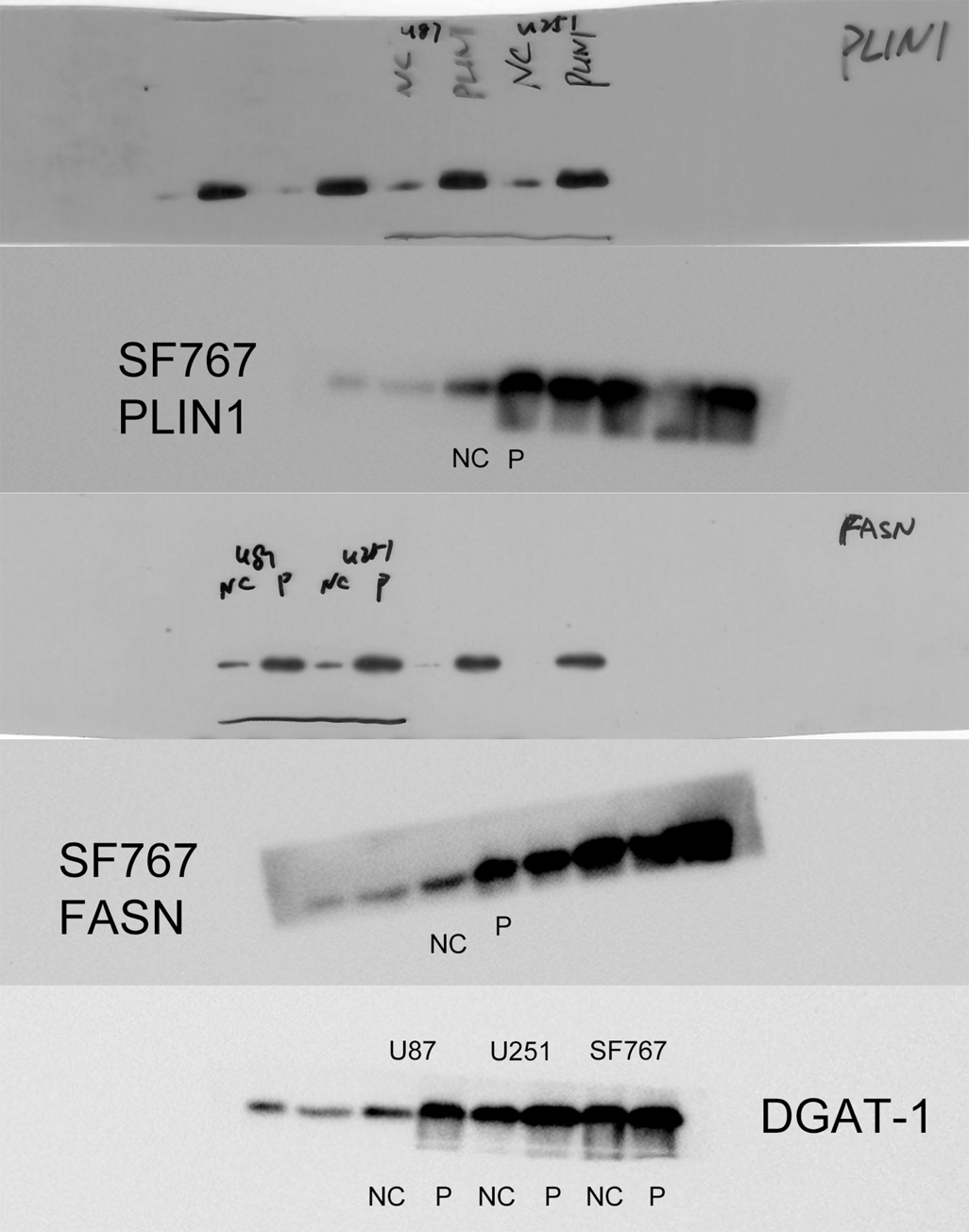


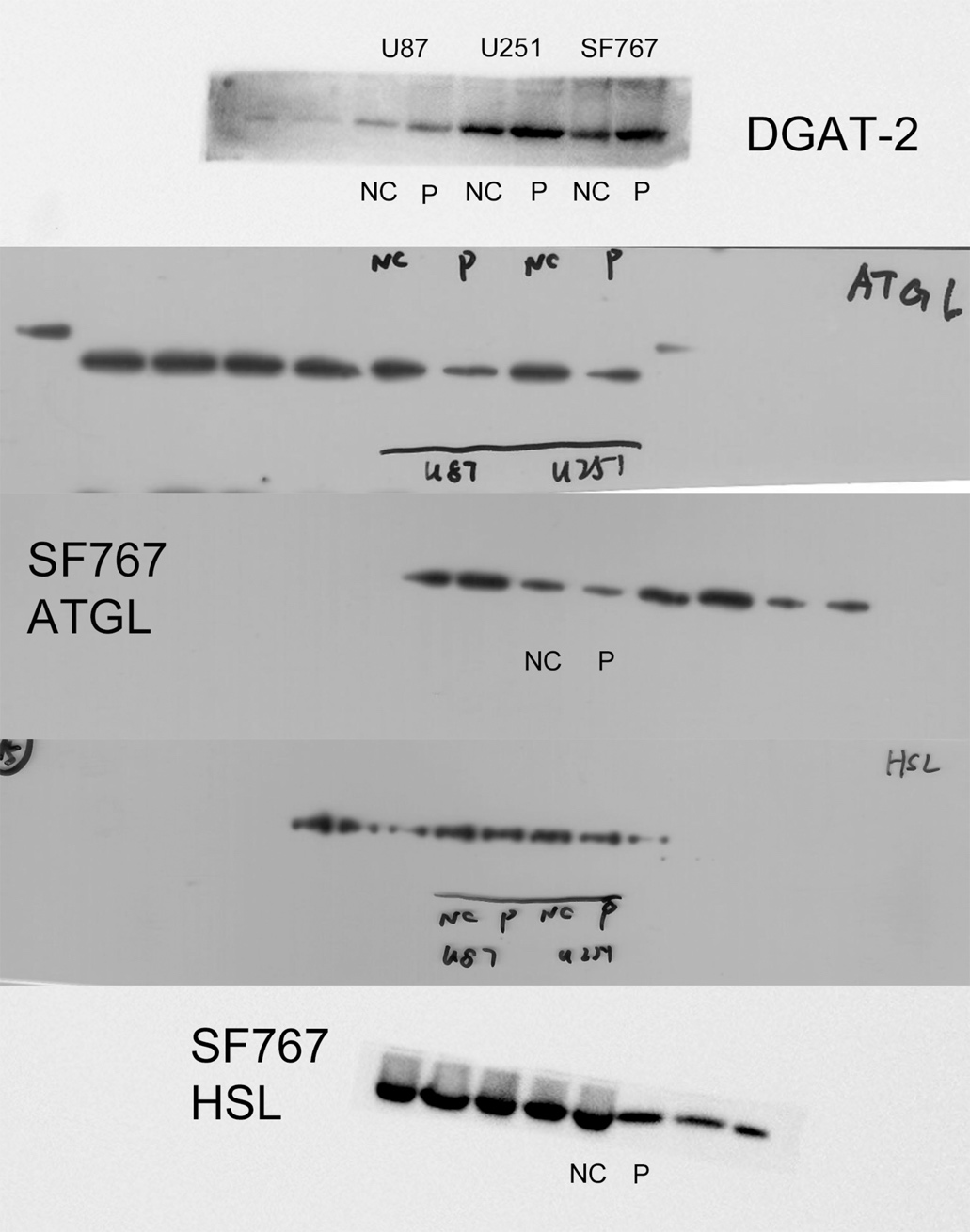


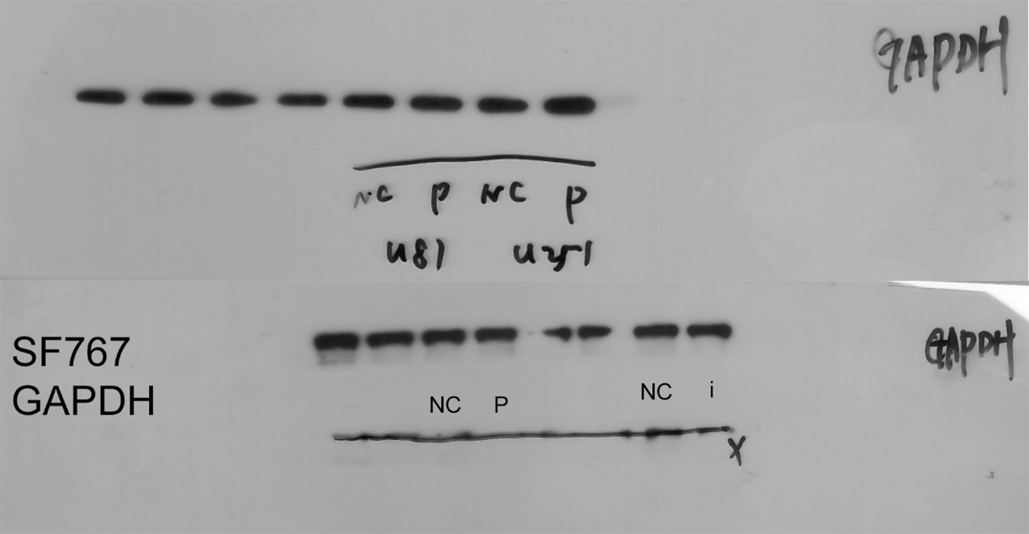


F5B


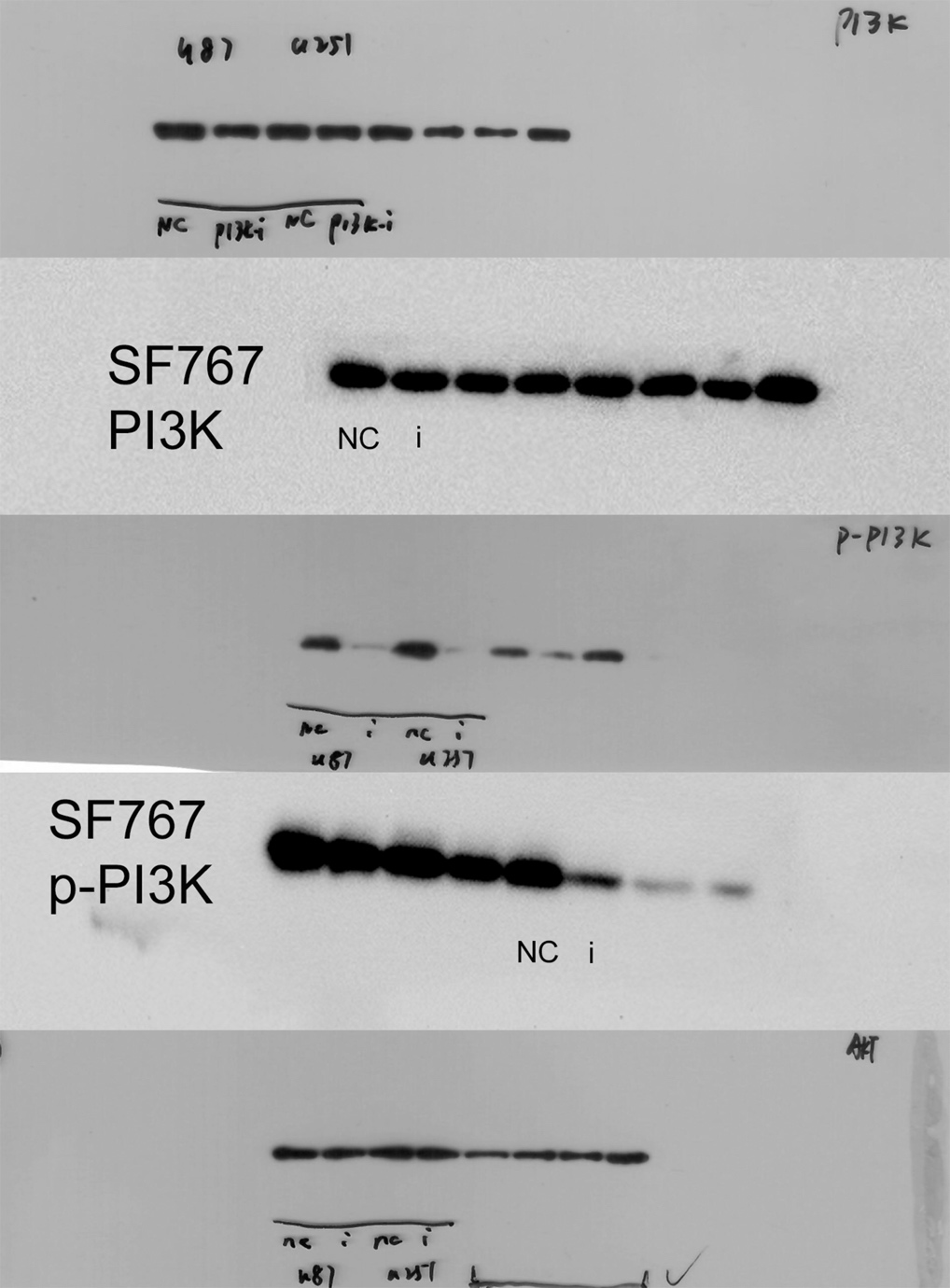


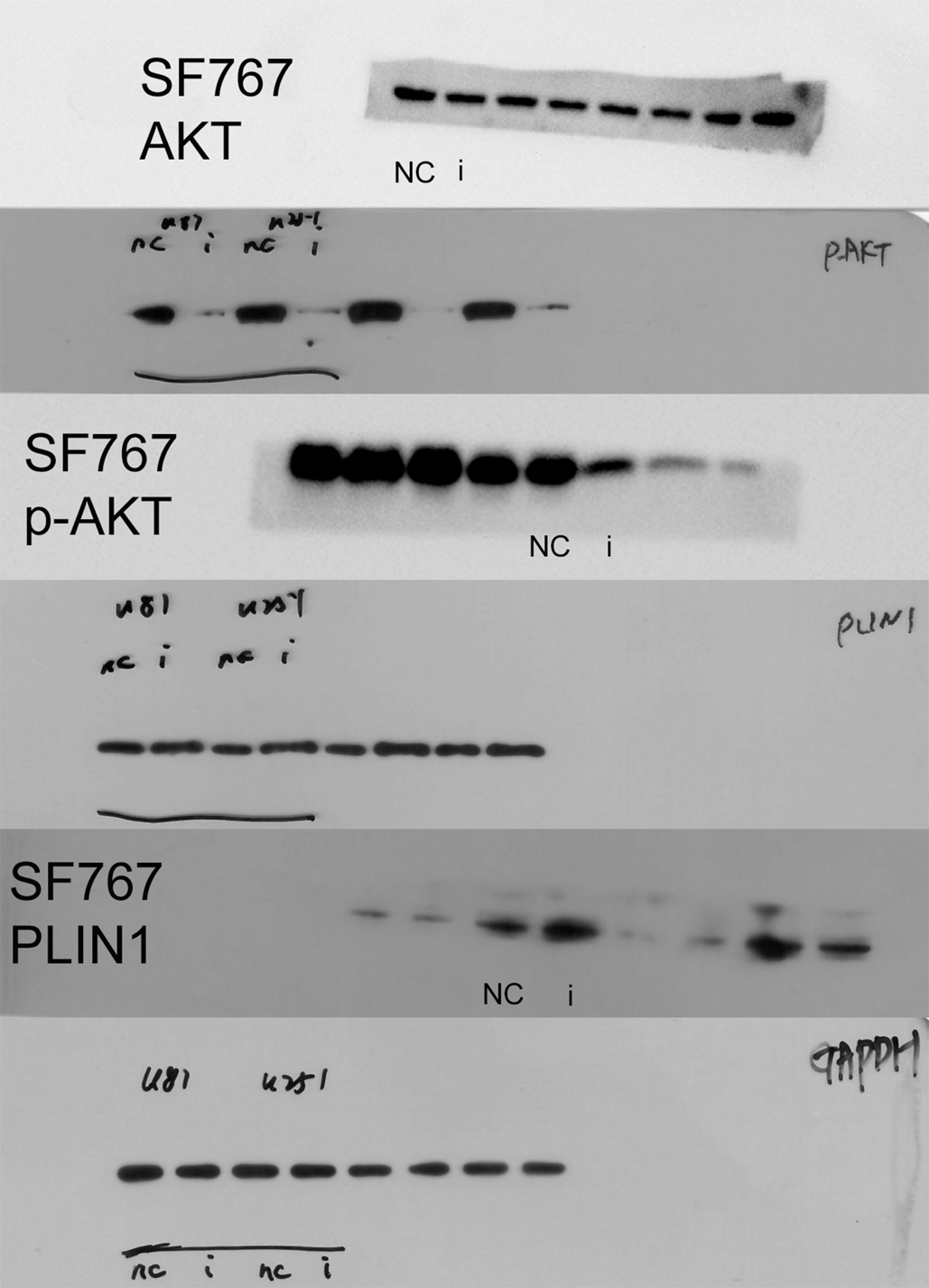


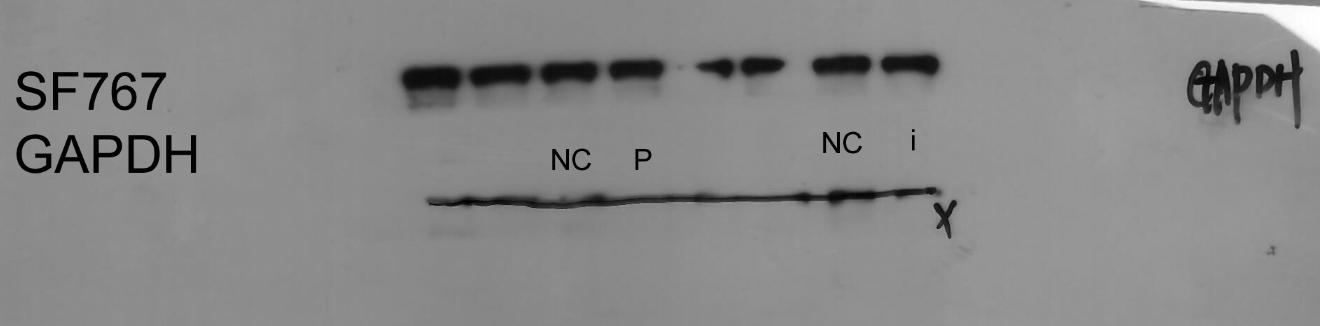

Supplement: Supplementary file 1 — Original western blots [file 41419_2025_7347_MOESM1_ESM.docx]
